# Metastatic and sentinel lymph node mapping using intravenously delivered Panitumumab-IRDye800CW

**DOI:** 10.7150/thno.55389

**Published:** 2021-05-24

**Authors:** Giri Krishnan, Nynke S. van den Berg, Naoki Nishio, Georgina Juniper, Jaqueline Pei, Quan Zhou, Guolan Lu, Yu-Jin Lee, Kimberly Ramos, Andrei H. Iagaru, Fred M. Baik, Alexander D. Colevas, Brock A. Martin, Eben L. Rosenthal

**Affiliations:** 1 Department of Otolaryngology - Division of Head and Neck Surgery, Stanford University School of Medicine, Stanford, CA, United States.; 2 Department of Otolaryngology, Head and Neck Surgery, The University of Adelaide, Adelaide, SA, Australia.; 3 Department of Radiology, Division of Nuclear Medicine, Stanford University School of Medicine, Stanford, CA, United States.; 4 Department of Medicine, Division of Medical Oncology, Stanford University School of Medicine, Stanford, CA, United States.; 5 Department of Pathology, Stanford University School of Medicine, Stanford, CA, United States.

**Keywords:** Head and neck cancer, Oral squamous cell carcinoma, sentinel lymph node biopsy, Fluorescent molecular imaging, Translational science

## Abstract

**Rationale:** Sentinel lymph node biopsy (SLNB) is a well-established minimally invasive staging procedure that maps the spread of tumour metastases from their primary site to the regional lymphatics. Currently, the procedure requires the local peri-tumoural injection of radiolabelled and/or optical agents, and is therefore operator dependent, disruptive to surgical workflow and restricted largely to a small subset of malignancies that can be readily accessed externally for local tracer injection. The present study set out to determine whether intravenous (IV) infusion of a tumor-targeted tracer could identify sentinel and metastatic lymph nodes (LNs) in order to overcome these limitations.

**Methods:** We examined 27 patients with oral squamous cell carcinoma (OSCC), 18 of whom were clinically node negative (cN0). Patients were infused intravenously with 50mg of Panitumumab-IRDye800CW prior to surgical resection of their primary tumour with neck dissection and/or SLNB. Lymphadenectomy specimens underwent fluorescence molecular imaging to evaluate tracer distribution to LNs.

**Results:** A total of 960 LNs were analysed, of which 34 (3.5%) contained metastatic disease. Panitumumab-IRDye800CW preferentially localized to metastatic and sentinel LNs as evidenced by a higher fluorescent signal relative to other lymph nodes. The median MFI of metastatic LNs was significantly higher than the median MFI of benign LNs (0.06 versus 0.02, p < 0.05).

Furthermore, selecting the highest five fluorescence intensity LNs from individual specimens resulted in 100% sensitivity, 85.8% specificity and 100% negative predictive value (NPV) for the detection of occult metastases and 100% accuracy for clinically staging the neck. In the cN+ cohort, assessment of the highest 5 fluorescence LNs per patient had 87.5% sensitivity, 93.2% specificity and 99.1% NPV for the detection of metastatic nodes.

**Conclusion:** When intravenously infused, a tumour-targeted tracer localized to sentinel and metastatic lymph nodes. Further validation of an IV tumor-targeted tracer delivery approach for SLNB could dramatically change the practice of SLNB, allowing its application to other malignancies where the primary tumour is not accessible for local tracer injection.

## Introduction

Head and neck cancer is a major cause of global morbidity and mortality 1. Oral squamous cell carcinoma (OSCC) is one of the most common types and has a high propensity to metastasise to cervical lymph nodes (LNs) 1, 2. Because the presence of cervical metastases is associated with reduced survival, management of the neck focuses on optimizing staging accuracy while minimizing treatment morbidity 3-7. This is challenging as occult metastases are present in 25% of patients who have negative clinical and radiographical staging (cN0) 4, 8-12. Currently the standard practice in the United States is to have cN0 patients undergo elective neck dissection (END) if there is a possibility (20%) that the LNs harbor micro-metastatic disease. This procedure consists of removal of the cervical fibrofatty lymphoid tissue in selected neck levels to evaluate for tumor positive LNs 13-15.

Sentinel lymph node (SLN) biopsy (SLNB) is an alternative method of identifying micrometastatic disease which is supported by the National Comprehensive Cancer Network (NCCN) guidelines 16, 17. This technique requires a local peri-tumoral radiotracer injection and assumes that the tracer follows the same route of lymphatic spread as metastatic cells. Subsequent excision and histopathological assessment of the SLN (the first LN receiving afferent flow of tracer) predicts the metastatic status of the neck 18-20. This technique offers lower morbidity than END with equal diagnostic yield, however it has not been adopted in the United States because it is dependent on appropriate local tracer injection, which can be disruptive to operative work flow, and is additionally restricted in the head and neck to those cancers arising in the oral cavity (since cancers arising from the pharynx and larynx cannot be accessed externally for local tracer injection) 21-27. Intravenous (IV) tracer delivery would overcome these limitations 28, 29.

Recently, our group demonstrated that IV infusion of a fluorescent antibody-dye conjugate (panitumumab-IRDye800CW) can detect metastatic LNs during pathological processing 30. Based on these findings, we hypothesized that IV panitumumab-IRDye800CW could potentially replace local tracer injection as a method for identification of SLNs during surgery. The present study aims to identify whether systemically delivered panitumumab-IRDye800CW can identify metastatic and sentinel LNs in resected neck dissection specimens by using the fluorescence signal of LNs to evaluate preferential tracer distribution.

## Methods

### Clinical trial design

Two prospective single centre, non-randomized, phase I-II clinical trials evaluating the fluorescence molecular imaging agent pantimumamab-IRDye800CW were approved by the Stanford University Administrative Panel on Human Subjects Research and the FDA and registered with ClinicalTrials.gov (NCT02415881; NCT03405142). The studies were performed in accordance with the Helsinki Declaration of 1975 and its amendments, FDA's ICH-GCP guidelines, and the laws and regulations of the United States. Written informed consent was obtained from all patients.

Between June 2018 and December 2019, adult patients with biopsy proven primary or recurrent OSCC scheduled for curative surgical treatment with a subsequent neck dissection (n = 25) or SLNB procedure (n = 2) were enrolled. Patients were divided into cN0 (clinically node negative (n = 18)) and cN+ (clinically node positive (n = 9)) cohorts based on consensus staging at the Stanford tumor board meeting after review of clinical findings and pre-operative imaging.

### Panitumumab-IRDye800CW

All patients received a flat dose of 50 mg of panitumumab IRDye800CW, infused via a peripheral IV catheter 1-5 days prior to surgery. Panitumumab (Vectibix; Amgen, Thousand Oaks, CA) is a recombinant, fully humanized monoclonal antibody, that binds with high affinity to the extracellular domain of the human epithelial growth factor receptor (EGFR), overexpressed in up to 90% of patients with HNSCC 31. IRDye800CW is a near infrared fluorophore, which is ideal for surgical visibility since it has higher tissue penetration depth than fluorescence in the visible range (400-700nm) and is not limited by endogenous autofluorescence 32. It has been demonstrated to have low toxicity and short half-life when unconjugated 33.

Panitumumab (Vectibix; Amgen; 147 kDa) was conjugated to IRDye800CW-NHS by a 2-hour incubation at 20C in the dark with a dye-to-protein ratio of 2.3:1. The N-hydroxysuccinimide (NHS) ester reaction binds randomly to lysines throughout the antibody during a relatively simple labelling method that has been performed successfully for chimeric and fully human antibodies with a consistent dye to protein ratio and good imaging results 34, 35. Quality control of the conjugate included analysis of drug product in a sterile vial for particulates and integrity of the sterilizing filter. Upon production and vialing, vials were transported to Stanford University (Stanford, CA) where they were stored at the Stanford Health Care Investigational Pharmacy prior to use 31. The safety profile and pharmacokinetics of the antibody-dye conjugate have been previously reported 36. Furthermore, the antibody-dye conjugate has been found to have a stability profile non-inferior to panitumumab alone 37.

### Intra-operative fluorescence imaging

**Figure 1:**
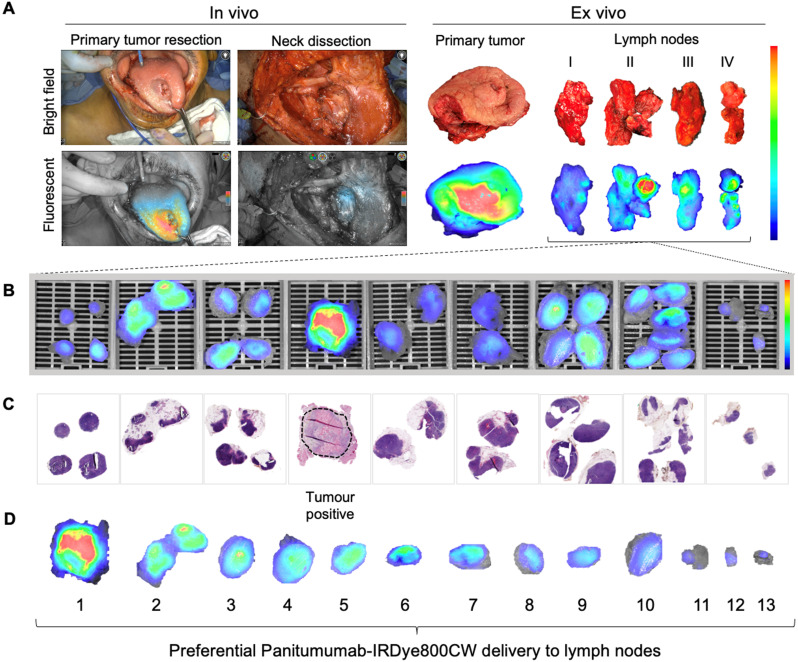
Lymph node fluorescence imaging workflow. **(A)** Representative case demonstrating intraoperative fluorescence imaging during resection of an anterior tongue tumour and elective level I - IV neck dissection. **(B)** Fluorescence image of cassetted LNs from the neck dissection specimen. **(C)** Corresponding H&E slides generated from the cassetted LNs enabling co-localisation of tumour status to fluorescence. **(D)** Ranking of individual LNs by mean fluorescence intensity to evaluate the preferential delivery and uptake of panitumumab-IRDye800CW to individual lymph nodes from the neck dissection specimen. LN: Lymph node, H&E: Haematoxylin and eosin.

The intraoperative fluorescence imaging workflow during END is shown in Figure 1A. Briefly, fluorescence imaging was performed before, during and after neck dissection using the Spy-Phi camera and the IR9000 optical imaging platform modified for IRDye800CW fluorescence imaging (Novadaq, Burnaby, Canada) 38, 39. A closed-field near-infrared optical imaging system ((modified) PEARL Triology, LI-COR Biosciences Inc.) was used on the back table for *ex vivo* imaging of surgical specimens 40, 41.

### *Ex vivo* fluorescence imaging and histopathology

Pathological processing and assessment of neck dissection specimens was conducted as standard of care and previously described 30. Cassetted tissue was re-imaged in the closed-field imaging device (LI-COR Biosciences) prior to paraffin embedding (Figure 1B). Stained slides were evaluated by a board-certified pathologist blinded to fluorescence. Tumor deposits were outlined and extranodal extension (ENE) was reported (Figure 1C). Selected slides underwent fluorescence microscopy as previously described 31.

### Sentinel lymph node biopsy

Two cN0 patients underwent conventional SLNB and IV infusion of panitumumab-IRDye800CW prior to END. In these patients, the afternoon prior to surgery, ^99m^Tc-tilmanocept (Lymphoseek, Cardinal Health, Dublin, OH, USA) was administered via 4 peri-tumoral injections of 0.125 mCi. Dynamic images were obtained during the first 10 minutes using a GE Discovery 870 CZT camera (GE Healthcare, Waukesha, WI, USA) followed by static planar images and SPECT/CT imaging. Images were evaluated by a board-certified nuclear medicine physician who reported the number and location of SLNs. Following primary tumor resection, a handheld gamma ray detection probe (Neoprobe, Johnson&Johnson Medical, via Leica Biosystems, Cincinnati, Ohio, United States) was used to track SLNs as seen on preoperative images. When localized, the count rate in SLNs was measured in triplicate. Next, fluorescence imaging was performed using a handheld near-infrared fluorescence camera (Spy-Phi, Novadaq). Identified SLNs were excised and completion neck dissection performed.

At pathology, SLNs were step-sectioned at 150 μm intervals to obtain slides from three levels. Routine immunohistochemistry was performed to confirm the presence of EGFR, cytokeratin 5/6, CD68, and CD31 using an autostainer (DAKO Link48 and PT link, Agilent Technologies Inc., Santa Clara, California, USA) as previously described 30. Stained slides were scanned digitally using a whole slide scanner (Hamamatsu NanoZoomer 2.0-RS, Hamamatsu, Japan). The presence of panitumumab-IRDye800CW was evaluated by imaging slide-mounted sections on the Odyssey imaging platform (LI-COR Biosciences Inc.).

### Statistical analysis

Mean fluorescence intensity (MFI), defined as total counts per pixel area, divided by pixel area, was calculated for each LN on acquired images using ImageStudio software (LI-COR Biosciences Inc.). MFI data was correlated to LN status (benign or metastatic) (Figure 1B and C). For LNs sent for intraoperative frozen section analysis, MFI was calculated from images acquired on the back table to avoid wash-out artefact as a result of altered tissue handling. MFI values for all LNs were entered into a spreadsheet (Microsoft Excel version 2019).

GraphPad Prism (version 8.0c) was used for statistical analysis. The Fisher exact test or chi-square test was used to compare categorical values between groups. The Mann-Whitney U-test was used to compare continuous values between groups and the MFIs between metastatic and benign LNs. Sensitivity and specificity of panitumumab-IRDye800CW to identify metastatic LNs was calculated using receiver operating characteristic (ROC) curves. The likelihood ratio was defined as sensitivity / (1 - specificity). All tests were two-sided. Data was presented as mean or mean ± standard deviation (SD), and a two-sided p-value of 0.05 or less was considered statistically significant (*p < 0.05; **p < 0.01; *** p < 0.001; NS not significant).

## Results

### Patient characteristics

Twenty-seven patients underwent IV infusion of panitumumab-IRDye800CW 1-5 days prior to END. There were 18 patients preoperatively staged as cN0 and 9 staged as cN+. There were no statistical differences in baseline demographics between groups (Table 1). Twenty-three unilateral and 4 bilateral neck dissections were performed, as well as two conventional SLNB procedures.

A total of 960 LNs were collected from neck dissections for analysis, of which 34 (3.5%) contained metastases. In the cN0 group, 10 out of 581 LNs (1.7%) were metastatic, and in the cN+ group 24 out of 379 LNs (6.3%) were metastatic. On average, 37 LNs were dissected per patient. Of the 18 patients with cN0 disease, five had metastatic disease at final pathology and were upstaged to pN1 in two (11.1%), pN2 in one (5.6%) and pN3 in two cases (11.1%). Of the 9 patients with cN+ disease, two (22.2%) had no metastases at final pathology and were down-staged to pN0 disease.

The majority (70.4%) of patients were infused within 2 days of surgery. Eight patients (4, 5, 6, 7, 11, 12, 15, 20) were infused the day before surgery and eleven patients (1, 2, 3, 9, 10, 13, 16, 18, 21, 23, 27) were infused 2 days prior to surgery. Three patients (14, 17, 22) were infused three days prior to surgery, and four patients (8, 24, 25, 26) were infused 4 days prior to surgery. Patient 19 was the only patient infused 5 days prior to surgery.

### Panitumumab-IRDye800CW distribution to metastatic and benign LNs

**Figure 2:**
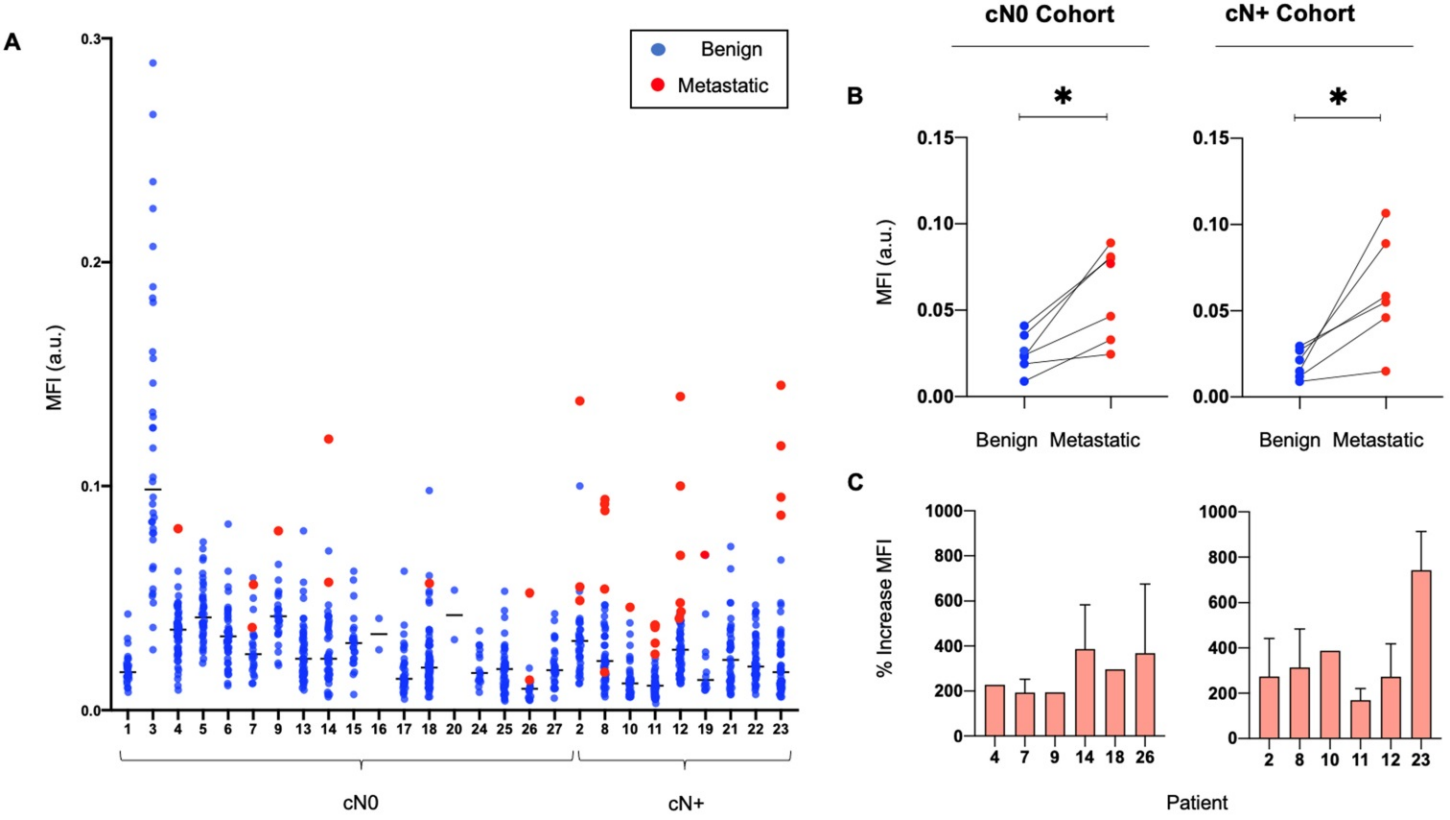
Metastatic LNs consistently exhibit a higher MFI than benign LNs in individual patients. **(A)** Graphical representation of the MFI of all resected metastatic and benign LNs per patient. **(B)** Median MFI of benign LNs versus median MFI of metastatic LNs per patient in both cN0 and cN+ patient cohorts. **(C)** Percentage increase in MFI of each resected metastatic LN above the median MFI of resected benign LNs per patient with pathologically positive neck disease. LN: Lymph node, MFI: Mean fluorescent intensity, cN0: Clinically node-negative, cN+: Clinically node-positive, Flu: Fluorescent image.

The median MFI of metastatic LNs was significantly higher than the median MFI of benign LNs (0.06 versus 0.02, p < 0.05). Ranking LNs by MFI in individual patients shows that metastatic LNs tend to exhibit a higher MFI than benign LNs (Figure 2A), with up to an 800% increase in signal between the median MFI of benign and metastatic LNs per patient (Figure 2C). This is statistically significant when comparing the fluorescence intensity of metastatic LNs to the fluorescence intensity of benign LNs within the same patients, in both the cN0 and cN+ cohorts (Figure 2B).

### Isolation of metastatic LNs based on preferential Panitumumab IRDye800CW uptake

**Figure 3:**
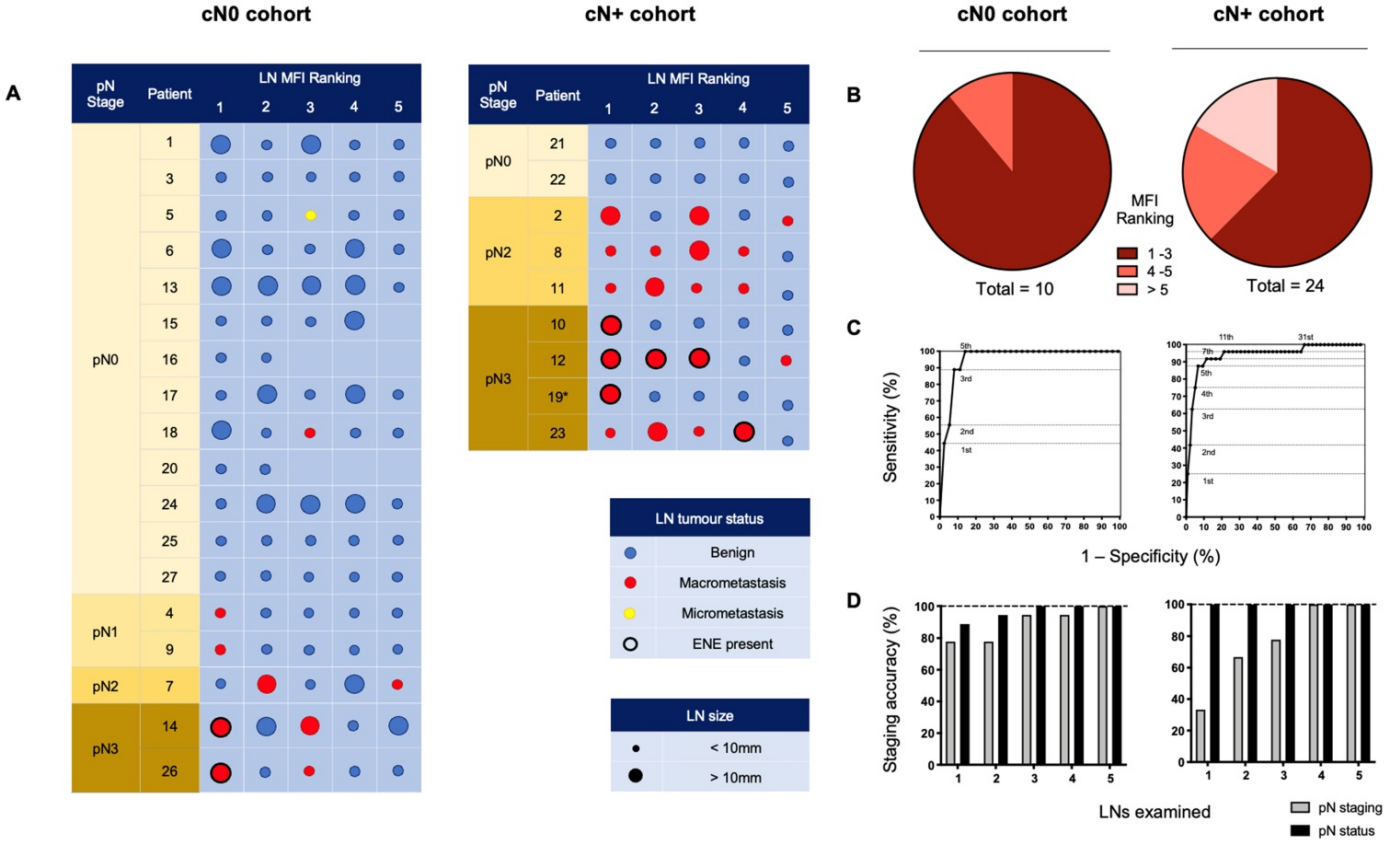
Ranking individually resected LNs by mean fluorescence intensity to isolate metastases and stage the neck. **(A)** The 5 LNs with the highest MFI from cN0 and cN+ patients, ranked from left to right by descending fluorescence signal demonstrating isolation of metastases. **(B)** Pie charts sorting metastatic LNs by relative fluorescent intensity ranking. **(C)** ROC curves based on MFI ranking of LNs. Assessment of the top 5 fluorescence LNs per patient, in the cN0 cohort; resulted in 100% sensitivity, 85.8% specificity and 100% negative predictive value (NPV), and in the cN+ cohort; 87.5% sensitivity, 93.2% specificity and 99.1% NPV, for the detection of (occult) metastatic nodes. **(D)** Accuracy of staging of the metastatic status and pathological status (according to the AJCC 8^th^ edition) of the necks across cN0 and cN+ cohorts based on the number of highest relative fluorescent intensity LNs assessed. LN: Lymph node, MFI: Mean fluorescent intensity, cN0: Clinically node-negative, cN+: Clinically node-positive, RFI: Relative fluorescent intensity, pN: Pathological node-stage, ENE: Extranodal extension.

All LNs from individual patient lymphadenectomy specimens were ordered by descending MFI providing an indication of preferential panitumumab-IRDye800CW delivery. To understand if this tracer could isolate metastatic LNs during a SLNB procedure, we examined the 5 LNs with the highest fluorescent intensity in each cohort (Figure 3A). Due to the anatomic complexity and variable lymphatic drainage in the head and neck, we chose to measure 5 LNs 42. Using this method in the cN0 group, 100% of patients with positive LNs could be identified. In fact, 9 out of 10 (90%) metastatic LNs ranked 1^st^, 2^nd^, or 3^rd^ in MFI relative all other LNs (Figure 3B). When evaluating cN+ patients, 16 out of 24 (66.7%) of the metastatic LNs ranked in the first 3 positions; 5 out of 24 (20.8%) ranked in the 4^th^ and 5^th^ positions; and 3 out of 24 (12.5%) ranked outside that range (Figure 3B).

ROC curves based on MFI ranking positions (Figure 3C) demonstrated that in the cN0 cohort, assessment of the highest 5 fluorescence LNs per patient allowed for 100% sensitivity, 85.8% specificity and 100% negative predictive value (NPV) for the detection of occult metastases. In the cN+ cohort, assessment of the highest 5 fluorescence LNs per patient had 87.5% sensitivity, 93.2% specificity and 99.1% NPV for the detection of metastatic nodes. Therefore, by setting a threshold of the highest 5 ranking LNs per patient for examination, in the cN0 cohort, of the 90 LNs examined, 80 were negative for tumour. In the cN+ cohort, of the 45 LNs examined, 24 were negative for tumour.

### Nodal Staging using Panitumumab IRDye800CW

To understand if IV panitumumab-IRDye800CW could accurately stage the cN0 neck, we performed pathological staging and assessment of the overall metastatic status of the neck based on the 5 highest MFI LNs from each patient and correlated these results with the final staging of complete lymphadenectomy specimens. In the cN0 cohort we found that analysis of only three LNs with highest tracer uptake allowed for accurate staging of the overall metastatic status of the neck in all cases (Figure 3D). To evaluate the potential clinical value of super-selecting high MFI LNs in the cN+ cohort, we found that examination of the highest four fluorescent LNs was all that was required in order to obtain accurate neck staging in each patient across the cohort (Figure 3D).

**Figure 4:**
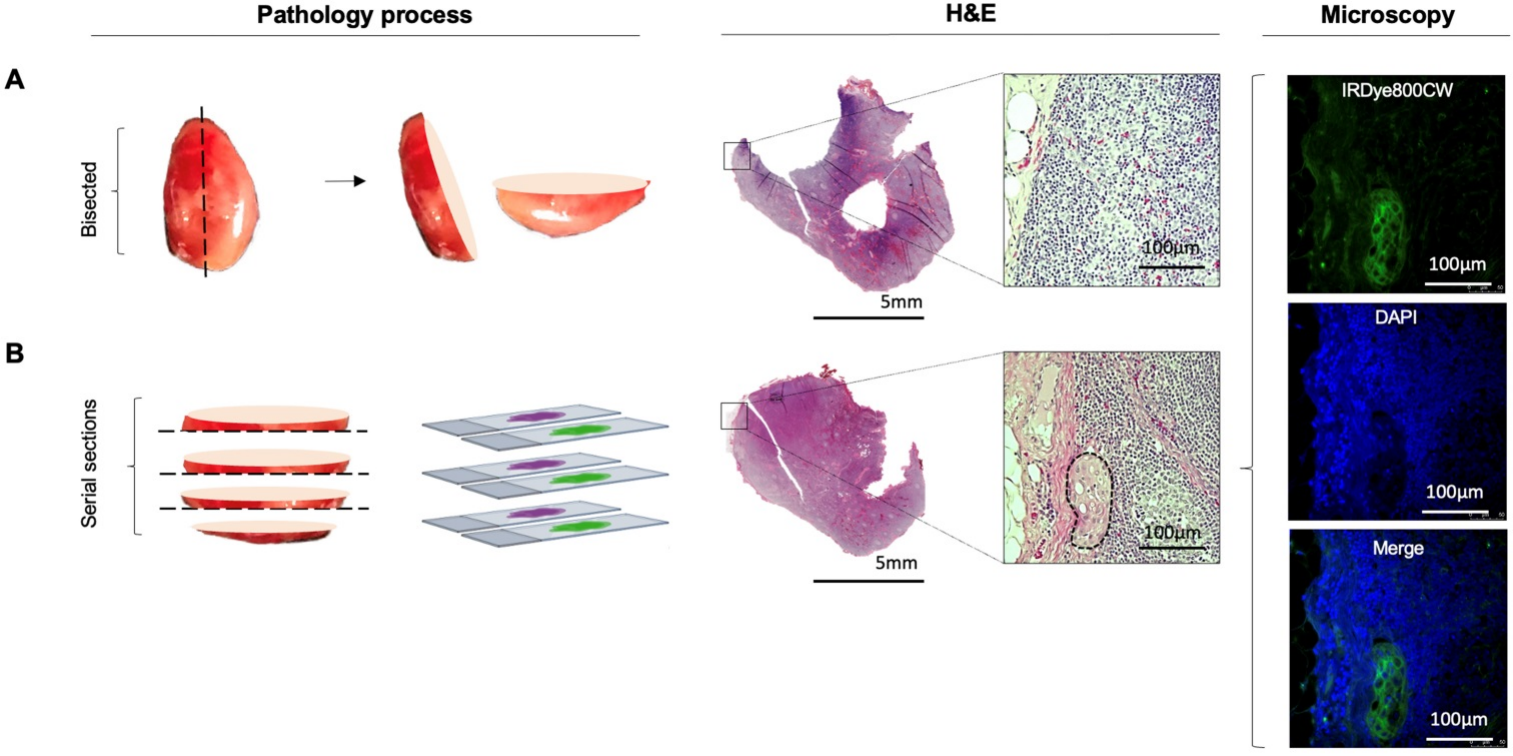
Further sectioning of LNs with high relative fluorescence can detect missed occult micrometastases. **(A)** Representative LN with high relative fluorescence, which upon routine histopathological processing showed no tumour deposit. **(B)** Further sectioning of this LN using a standard SLNB pathological protocol revealed a missed microscopic tumour deposit. Microscopy fluorescence imaging demonstrates clear colocalization of fluorescent intensities at 800nm with uniform, strong cytoplasmic and membranous binding of IRDye800CW to the tumour cells, not observed in normal tissue as seen following DAPI staining of the nuclei of the surrounding tissue. LN: Lymph node, SLNB: Sentinel lymph node biopsy, H&E: Haematoxylin and eosin, DAPI: 4',6-diamidino-2-phenylinodole.

As routine histopathology misses micrometastases, a SLNB pathology protocol was also performed on the highest three fluorescent LNs from cN0 patients (Figure 4) 14, 43. Chance-sampling error was controlled for by examining size-matched LNs from the same patient in a blinded fashion. One micro-metastasis was revealed as a false negative (Figure 4), which upgraded the stage in one patient from pN0 to pN1. None of the control LNs were positive for metastases.

### Comparison of IV Panitumumab IRDye800CW with peri-tumoral ^99m^Tc-tilmanoscept

**Figure 5:**
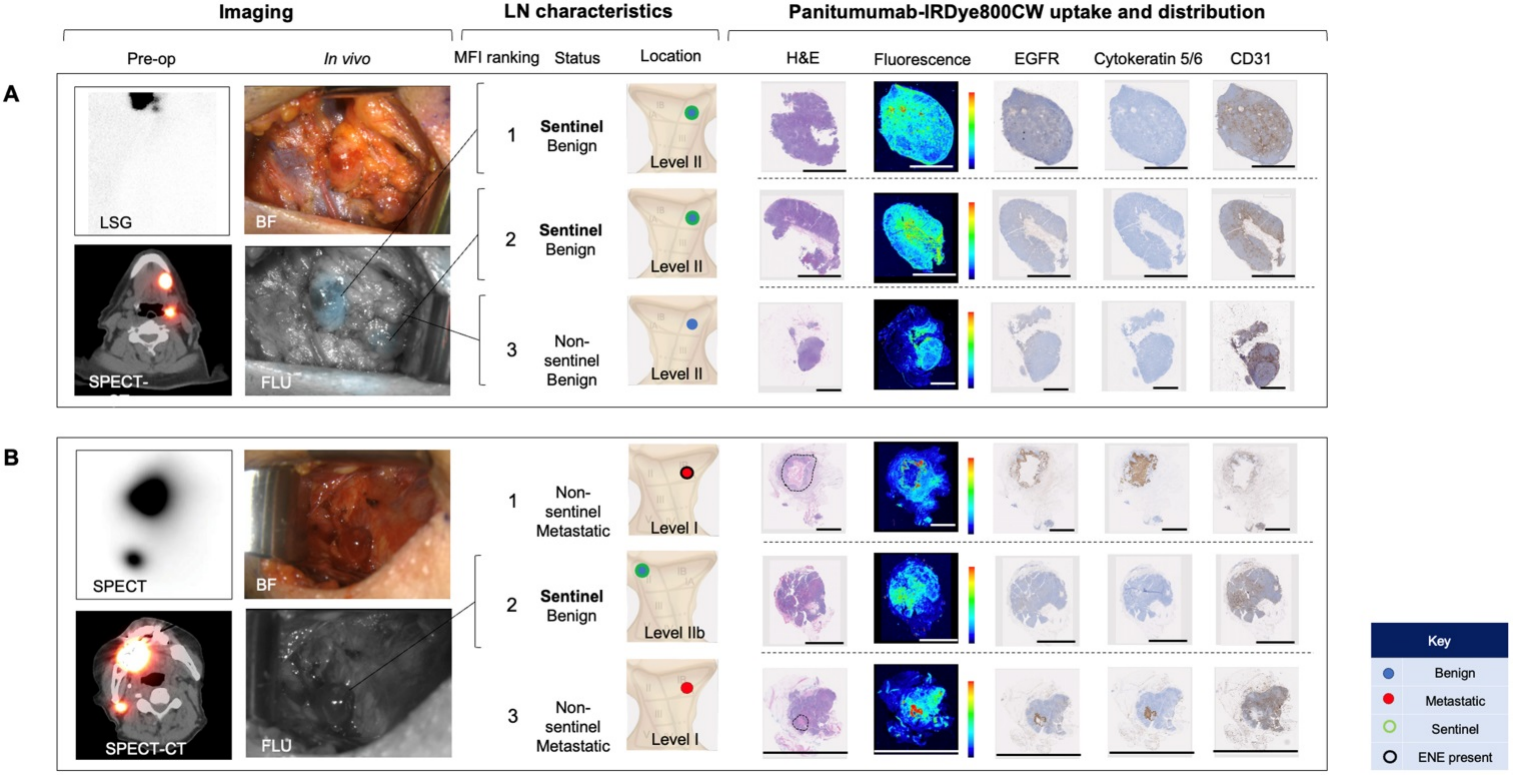
Systemically delivered panitumumab-IRDye800CW demonstrates high relative fluorescence in sentinel lymph nodes. **(A)** Case A demonstrates a patient with two level II SLNs following local radiotracer injection. These were the highest two fluorescent LNs following completion END. H&E staining showed no tumour deposits in these LNs and fluorescence imaging showed signal throughout the subcapsular and trabecular sinuses with minimal EGFR expression.** (B)** Case B demonstrates a patient with one level II SLN. This was the second highest fluorescent LN following completion END. Fluorescence imaging and IHC showed a similar distribution pattern of panitumumab-IRDye800CW to the SLNs in case A. The first and third highest fluorescent LNs in this patient were metastatic (tumour deposit outlined on H&E). Fluorescence in these LNs correlated strongly with EGFR expression. In the 12mm metastasis with ENE, fluorescence was highest around the periphery of the deposit. In the smaller metastasis, fluorescence was well distributed throughout the tumour deposit. Fluorescence also corelated strongly with CD31 staining in both metastatic LNs. Scale bars = 100 μm. SLN: Sentinel lymph node, END: Elective neck dissection, H&E: Haematoxylin and eosin, IHC: Immunohistochemistry, EGFR: Epithelial growth factor receptor, LSG: Lymphoscintigraphy, SPECT: single photon computed tomography, SPECT-CT: single photon computed tomography and computed tomography (SPECT/CT) FLU: Fluorescent image.

To evaluate whether IV panitumumab-IRDye800CW isolates SLNs identified with peri-tumoral injection of ^99m^Tc-tilmanoscept, two patients received both IV panitumumab-IRDye800CW and peri-tumoral ^99m^Tc-tilmanoscept. SLNs receiving direct ^99m^Tc-tilmanoscept drainage were identified with preoperative radionuclear imaging (Figure 5) 18.

In the first case (Case A), using a conventional SLNB technique, a cluster of two SLNs were identified in level II of the left neck; in the second case (Case B) one SLN was identified in level II of the right neck. Intraoperatively, all SLNs were successfully identified using a handheld gamma ray detection probe. In Case A (Figure 5A), the cluster of two SLNs were identified and registering gamma readings averaging 3475 and 3560, respectively. *In vivo* fluorescence imaging demonstrated signal in both of these SLNs (with MFIs of 0.043 and 0.040 respectively). In Case B (Figure 5B) the SLN recorded an average gamma reading of 1695 and was poorly visualized with the open-field near-infrared fluorescence camera and had an MFI of 0.019. In both patients an END was performed, and none of the SLNs contained metastatic disease on final pathology. However in Case B, two LNs on final pathology, that were not identified by ^99m^Tc-tilmanoscept, were found to contain metastatic disease. These two metastatic LNs had the first and third highest MFI among all LNs from the specimen (MFIs of 0.052 and 0.019 respectively). This suggests that the injection may have been performed in a manner which did not accurately identify the SLN. In Case A, the two SLNs identified by conventional methods, were registered to have the highest fluorescence signals (Figure 5A).

We evaluated the microscopic distribution of panitumumab-IRDye800CW within the histological architecture of the LNs. Lymph nodes that were negative for metastases demonstrated florescent signal throughout the subcapsular and trabecular sinuses in the absence of EGFR expression. There was strong correlation of fluorescence with the microvasculature based on CD31 staining. On the other hand, the fluorescence signal within metastatic LNs correlated strongly with EGFR expression. The microscopic distribution of fluorescence in benign LNs and metastatic LNs was not significantly changed depending on whether the LN was sentinel or non-sentinel.

## Discussion

Since its description in 1977, the SLNB procedure has depended on peri-tumoural delivery of non-targeted agents 19. To the best of our knowledge, this is the first study to identify a systemically delivered, SLN molecular agent in humans. By analyzing fluorescence signal within individual LNs from resected lymphadenectomy specimens, we found that panitumumab-IRDye800CW identifies at-risk (metastatic and/or sentinel) LNs, allowing for accurate staging of cN0 patients.

We previously demonstrated in a dose escalation study, that fluorescence imaging of LNs at pathology of patients preoperatively infused with IV panitumumab-IRDye800CW enabled discrimination of metastatic and benign LNs based on a signal-to-background ratio (LN MFI divided by background tissue MFI) 30. In the present study, we evaluated the feasibility of IV infusion of a molecularly-targeted fluorescent tracer to identify LNs most likely to harbour occult metastatic disease. We demonstrated that panitumumab-IRDye800CW preferentially reached SLNs, regardless of the tumor status of the LN. We demonstrated in two patients who underwent conventional SLNB, that IV panitumumab-IRDye800CW was preferentially delivered to the same LNs identified by ^99m^Tc-tilmanoscept as being the first draining LNs from the primary tumor. Furthermore, identification of tumour-positive LNs based on preferential tracer uptake was not impeded by the presence of occult metastases within other LNs, which have potential to obstruct flow of locally delivered tracer, providing further support of this novel approach in the anatomically complex head and neck region.

The mechanism by which a systemically delivered agent identifies the SLNs is unclear, but it is possible that the antibody-dye bioconjugate first accumulates in the primary tumor and then drains non-specifically into the regional nodal basin in a stepwise pattern. It is likely that metastatic and sentinel LNs may be more likely to retain the panitumumab-IRDye800CW given the relatively large size (roughly 150kD) of the molecular tracer.

There are several key differences between this study and the study by Nishio et al. Firstly, the study by Nishio et al was a dose-escalation study where patients were divided into three different cohorts and received different weight-based doses of panitumuanmb-IRDye800CW. In the present study a fixed dose of 50 mg was used for all patients. Furthermore, to account for differences in tumour heterogeneity and regional nodal spread, only patients with oral squamous cell carcinoma were included in this study, while all head and neck squamous cell carcinoma patients were included in Nishio's study. Finally, and most importantly, Nishio et al used a fixed thresholds for MFI and SBR to discriminate positive and negative LNs, whereas in the present study, LNs were ranked by decreasing order of MFI per patient and accuracy of positive tumour detection was calculated based on how many of the highest ranked LNs were needed. In both studies, MFI-based ranking of dissected LNs was used to identify how many LNs needed to be examined in order to accurately stage the neck. In our study 5 LNs from the cN0 group and 4 LNs from the CN+ group were required to achieve 100% accuracy with staging. In the study by Nishio et al, only 3 LNs were required across all patients to achieve 100% accuracy of staging.

Because all the patients underwent complete removal of all the lymph nodes, we were able to identify all the sentinel and metastatic LNs through fluorescent analysis of all LNs. This study examined mostly cN0 patients, while inclusion of cN+ patients increased the number of metastatic LNs for analysis. All patients received the same dose of 50 mg of panitumumab-IRDye800CW, which is the established optimal dose for intra-operative imaging 44. A notable limitation was that only two patients underwent conventional SLNB prior to END resulting in a small sample size for this direct comparison. In this study, patients were infused 1-5 days prior to surgery with panitumumab-IRDye800CW, which has been consistent with other trials conducted by our group studying this drug. Ideally, the timing of infusion prior to surgery would be kept constant, as our group have previously demonstrated that MFI significantly increased when the infusion-to-surgery window was reduced to within 2 days compared with 3 days or more 45, however this can be logistically challenging, and is subject to patient appointment availability and scheduling of surgery.

In this study, because of interpatient heterogeneity there was overlap in MFI between benign and malignant LNs over the population studied. Other groups have used techniques to improve cancer-specific discrimination, such as the radiometric imaging work by Ngyuen et al., where quantitative discrimination between target tissues and background based on the ratio of two fluorophores (rather than one) with intensities at different wavelengths, controls for variability in factors such as the amount of probe administered, patient-specific pharmacokinetics and imaging parameters 46-48. Another example is the paired-agent molecular imaging method used by Ntziachristos and Pogue, where the kinetics of a control imaging agent is used to account for the nonbinding related kinetics of a cancer-targeted imaging agent 49, 50. Unfortunately significant additional regulatory requirements and costs have limited our ability for the moment to apply these techniques clinically.

This study in a clear demonstration that a molecular imaging agent can be administered systemically to identify metastatic and sentinel LNs. However, implementation will require conjugation of panitumumab to an appropriate gamma emitting radiotracer to enable dual-modality surgical navigation, which has been established as the standard of care for non-targeted locally injected fluorescent tracers 51, 52. As is current practice, the antibody-radiolabel bioconjugate could identify at-risk LNs pre-operatively on SPECT/CT or PET/CT and then enable intraoperative localization with a gamma-probe. The conjugated fluorescent dye would allow for optical imaging with NIR-cameras to identify and isolate the node during dissection 53, 54. Consistent with our previous work, this could also identify non-sentinel metastatic LNs in a positive neck 30.

By using fluorescence intensity to evaluate the delivery of panitumumab-IRDye800CW to individual LNs in lymphadenectomy specimens, we provide evidence that systemic delivery of this tumour-targeted tracer can identify sentinel and metastatic LNs. This study therefore demonstrates feasibility of SLN mapping using a systemically delivered tumour-targeted tracer, and provides impetus for further trials evaluating this strategy in lung, oesophageal, colorectal, urological and gynaecologic malignancies, using a different appropriately targeted antibody. Broad translation of this novel approach when combined with radioguidance holds promise to expand the potential role of SLNB in oncological surgery.

## Figures and Tables

**Table 1:** Patient, primary tumour and lymph node characteristics.

Variable	Clinical LN status		P - value	Total
	cN0	cN+		
**Patient characteristics**				
Number of patients, No. (%) 9/.	18 (100)	9 (100)	0.10 **^c^**	27 (100)
Gender			0.67 **^b^**	
Female	7 (38.9)	3 (33.3)		10 (37.0)
Male	11 (61.1)	6 (66.7)		17 (63.0)
Age, mean (SD), y	63.2 (9.5)	60 (11.3)	0.53 **^c^**	62.2 (10.1)
Weight, mean (SD), kg	67.6 (15.8)	78.1 (11.9)	0.06^**c**^	71.1 (15.3)
Days from infusion to surgery, No. (%)			0.66 ^a^	
1	6 (33.3)	2 (22.2)		8 (29.6)
2	7 (38.9)	4 (44.4)		11 (40.7)
3	2 (11.1)	1 (11.1)		3 (11.1)
4	3 (16.7)	1 (11.1)		4 (14.8)
5	-	1 (11.1)		1 (3.7)
**Primary tumour characteristics**				
Tumour sub-site, No. (%)			0.83 ^a^	
Tongue	7 (38.9)	5 (55.6)		16 (59.3)
Buccal mucosa	6 (33.3)	3 (33.3)		9 (33.3)
Retromolar trigone	3 (15.8)	1 (11.1)		4 (14.8)
Alveolar ridge	1 (5.6)	-		1 (3.7)
Hard palate	1 (5.6)			1 (3.7)
Final T stage, No. (%)			0.10^ a^	
pTx	-	1 (11.1)		1 (3.7)
pT1	5 (27.8)	-		5 (18.5)
pT2	6 (33.3)	1 (11.1)		7 (25.9)
pT3	3 (15.8)	4 (44.4)		7 (25.9)
pT4	4 (22.2)	3 (33.3)		7 (25.9)
Recurrence, No. (%)	5 (27.8)	1 (11.1)		6 (22.2)
Maximal dimeter, mean (SD), cm	2.8 (1.5)	4.6 (2.3)	*0.03 **^c^**	3.4 (2.0)
Depth of invasion, mean (SD), mm	9.5 (7.5)	26.6 (22.1)	*0.02 **^c^**	15.8 (16.4)
**Lymph node characteristics**				
Lymph nodes (LNs), No. (%)			**0.001^ a^	
Total	581 (100)	379 (100)		960 (100)
Mean LNs per patient	33.9	42.1		37
Metastatic	10 (1.7)	24 (6.3)		34 (3.5)
Benign	571 (98.3)	355 (93.7)		926 (96.5)
Extra nodal extension (ENE), No. (%)				
Number of nodes	2 (0.3)	5 (1.3)		7 (0.7)
Patients with ENE	2 (11.1)	4 (44.4)		6 (22.2)
Clinical N-stage, No. (%)			****<0.0001^ a^	
N0	18 (100)	-		18 (66.7)
N1	-	3 (33.3)		3 (11.1)
N2	-	4 (44.4)		4 (14.8)
N3	-	2 (22.2)		2 (7.4)
Pathological N-stage, No. (%)			*0.02^ a^	
N0	13 (72.2)	2 (22.2)		15 (55.6)
N1	2 (11.1)	-		2 (7.4)
N2	1 (5.6)	3 (33.3)		4 (14.8)
N3	2 (11.1)	4 (44.4)		6 (22.2)
Neck dissection type, No. (%)			0.9 **^b^**	
Unilateral neck dissection	17 (94.4)	6 (66.7)		23 (85.2)
Bilateral neck dissection	1 (5.6)	3 (33.3)		4 (14.8)
Pre-operative imaging modality, No. (%)			0.12^ a^	
CT	4 (22.2)	1 (11.1)		5 (18.5)
PET/CT alone	-	3 (33.3)		3 (11.1)
MRI	7 (38.9)	3 (33.3)		10 (37.0)
PET/CT and MRI	4 (22.2)	2 (22.2)		6 (22.2)
*Unknown*	2 (11.1)	-		2 (7.4)

Chi-squared test^a^, Fisher's exact test^b^, Mann-Whitney test^c^.

## Abbreviations

END: elective neck dissection
EGFR: epithelial growth factor receptor
ENE: extranodal extension
FDA: food and drug administration
HNSCC: head and neck squamous cell carcinoma
IV: intravenous
LN(s): lymph nodes
MFI: mean fluorescence intensity
NCCN: national comprehensive cancer network
cN0: clinically negative nodal status
NPV: negative predictive value
OSCC: oral squamous cell carcinoma
ROC: receiver operating characteristic
SLN: sentinel lymph node
SLNB: Sentinel lymph node biopsy
SPECT: single photon emission computed tomography
SD: standard deviation
